# Thermomechanical Devulcanization of Highly Filled EPDM Rubber Waste: Process Optimization, Selectivity Assessment, and Preliminary Industrial Scale-Up

**DOI:** 10.3390/ma19214559

**Published:** 2026-10-29

**Authors:** Pedro Veiga Rodrigues, Mateus Vieira Ribeiro, Ana Vera Machado

**Affiliations:** Department of Polymer Engineering, Institute for Polymers and Composites (IPC), University of Minho, 4804-533 Guimarães, Portugal; pedro.rodrigues@dep.uminho.pt (P.V.R.); mateusvieiraribeiro.23@gmail.com (M.V.R.)

**Keywords:** EPDM waste, thermomechanical devulcanization, DBD, HDA, devulcanization scale-up

## Abstract

**Highlights:**

- Highly filled industrial EPDM waste was devulcanized by thermomechanical processing using DBD and HDA.
- Laboratory-scale devulcanization degrees of up to 86% were achieved under optimized conditions.
- DBD showed optimum performance at 1 phr and 200 °C, while higher loading reduced efficiency.
- HDA reached comparable devulcanization only at higher temperature and concentration.
- Industrial scale-up achieved 60% devulcanization but showed reduced selectivity, highlighting the requirement for improved mixing and residence-time control.

**Abstract:**

Devulcanization of ethylene–propylene–diene monomer (EPDM) rubber is a promising strategy to improve the circularity of vulcanized automotive rubber waste. However, selective sulfur crosslink cleavage while preserving the polymer backbone remains challenging, particularly in highly filled industrial compounds. In this work, highly filled sulfur-cured EPDM waste from automotive sealing-profile production was thermomechanically devulcanized using 2,2′-dibenzamidodiphenyl disulfide (DBD) and hexadecylamine (HDA). Laboratory-scale experiments were performed in an internal mixer, followed by preliminary industrial-scale trials in a single-screw extruder. Crosslink density was determined using the Flory–Rehner equation with Kraus correction, and selectivity was assessed by Horikx analysis, supported by ATR-FTIR and rheometry. At laboratory scale, DBD showed optimal performance at 1 phr and 200 °C, reaching approximately 86% devulcanization, while higher DBD loading reduced efficiency. HDA reached approximately 85% devulcanization at 3 phr and 280 °C. Horikx analysis indicated preferential crosslink scission under optimized conditions, although mixed reclamation behavior was still observed. Industrial scale-up with 1 phr DBD at 200 °C achieved approximately 60% devulcanization, while HDA was less effective. The lower performance observed at the industrial scale highlights the challenges of translating laboratory-optimized conditions to industrial processing, although the individual contributions of specific processing parameters could not be determined from the available data.

## 1. Introduction

The growing global consumption of vulcanized elastomers, particularly in the automotive sector, has generated large amounts of end-of-life rubber waste. This creates significant environmental and waste-management challenges [1]. Conventional disposal routes, such as landfilling, incineration, and pyrolysis, are increasingly restricted by European legislation and do not fully support circular economy principles, since they limit material recovery [2,3]. As a result, the rubber industry has been developing sustainable recovery strategies to reduce material costs, decrease the use of natural resources, and increase the recyclability of end-of-life vehicles to at least 95% by weight [4,5].

Ethylene–propylene–diene monomer (EPDM) rubber is widely used in automotive applications, including sealing profiles, weather strips, hoses, and vibration dampers. Its use is mainly due to its excellent resistance to heat, ozone, ultraviolet radiation, and weathering [6]. EPDM is a terpolymer composed of ethylene and propylene units in the main chain and a non-conjugated diene comonomer, such as 5-ethylidene-2-norbornene (ENB), dicyclopentadiene (DCPD), or 5-vinyl-2-norbornene (VNB), incorporated in the side chain. These diene units provide unsaturation sites for sulfur vulcanization [7]. However, the three-dimensional network formed during vulcanization makes EPDM difficult to reprocess using conventional polymer recycling methods. Therefore, recycling is often limited to mechanical grinding or energy recovery [8].

Among the recycling methods proposed for vulcanized rubbers, devulcanization is one of the most promising. It aims to selectively cleave sulfur crosslinks, such as C–S and S–S bonds, while preserving the polymer backbone. This approach can restore partial processability and allow the material to be reintroduced into new rubber formulations [9,10,11]. Thermomechanical devulcanization is particularly attractive because it can be performed using equipment already common in the rubber industry, such as internal mixers and extruders [12].

A major challenge in devulcanization is selectivity. The bond dissociation energies of S–S, C–S, and C–C bonds are relatively close, approximately 270, 310, and 370 kJ mol^−1^, respectively [13,14,15]. Under combined heat and shear, sulfur crosslink cleavage may therefore occur together with polymer backbone scission. When backbone degradation occurs, the process is better described as reclamation rather than selective devulcanization. This reduces molecular weight and can impair the mechanical properties of revulcanized materials [16].

To evaluate the selectivity of rubber devulcanization, Horikx developed a theoretical framework relating the decrease in crosslink density to the increase in soluble fraction [17]. This method distinguishes between random main-chain scission and selective crosslink scission [18,19]. It is particularly useful for sulfur-vulcanized EPDM, where the presence of stable monosulfidic crosslinks can make selective cleavage more difficult [20]. Therefore, Horikx analysis helps determine whether a given process promotes true devulcanization or less selective degradation.

The efficiency of thermomechanical devulcanization can be improved by using chemical devulcanizing agents. These agents act through different mechanisms, including radical stabilization and nucleophilic attack on sulfur-crosslink bonds [21]. Among the agents used for EPDM, 2,2′-dibenzamidodiphenyl disulfide (DBD) and hexadecylamine (HDA) have shown promising results. DBD is an organic disulfide that promotes crosslink cleavage through a radical mechanism and produces less sulfur odor than many alternative disulfides. Under heat and shear, DBD radicals promote cleavage and exchange reactions between S–S and C–S bonds, while excessive radical formation may favor recombination or partial re-crosslinking [2,22]. Conversely, HDA is an aliphatic primary amine that acts mainly through a nucleophilic attack on sulfur atoms, promoting cleavage of S–S and C–S bonds within the crosslinked network, and is effective against sulfide crosslinks [23]. From a processing perspective, DBD is advantageous because it achieves high devulcanization efficiency at lower temperature and concentration. Although HDA provides effective sulfur-crosslink cleavage, it requires higher loading, temperature, and adequate mixing and residence time. Therefore, DBD appears more attractive for industrial processing, while HDA may be useful in processes where more severe and well-controlled conditions can be maintained.

Despite these advances, several gaps still limit the industrial purpose of EPDM devulcanization. Most published studies about EPDM devulcanization use controlled compounds or tire-derived rubber, which do not fully represent highly filled industrial EPDM waste streams [23,24]. Gschwind et al. [24,25] worked with comparatively well-defined EPDM compounds, Dijkhuis [20] and Verbruggen et al. [3,18,23] used purpose-prepared EPDM vulcanizates, while Seghar et al. [9,19] focused on validating the Horikx framework using less heavily filled systems. Colom et al. [7] examined roofing-membrane EPDM, which differs substantially from automotive sealing formulations in both filler type and crosslink architecture. High filler contents, particularly carbon black and calcium carbonate, difficult the measurement of crosslink density since fillers restrict swelling. Therefore, correction methods such as the Kraus correction are required to avoid biased results [26]. In this work, industrial automotive sealing waste is used to assess the feasibility of EPDM devulcanization under industrial conditions and to compare the results with laboratory-scale experiments. To the best of our knowledge, direct comparisons between DBD and HDA for highly filled EPDM waste are still lacking. Previous studies have typically investigated these agents individually or employed different base compounds, preventing a direct comparison of their devulcanization mechanisms [2,24,25,27]. The transfer of optimized laboratory conditions to industrial-scale processes remains insufficiently documented.

Therefore, this work investigates the thermomechanical devulcanization of highly filled sulfur-vulcanized EPDM waste derived from automotive production non-conformities using DBD and HDA as devulcanizing agents, from laboratory to industrial conditions. The novelty of this study lies in three main aspects: (i) direct comparison of DBD (a radical mechanism) and HDA (a nucleophilic mechanism), within the same highly filled industrial EPDM waste matrix; (ii) application of a combined analytical framework based on Flory–Rehner swelling analysis with Kraus correction, Horikx analysis, ATR-FTIR, and rheometry to assess network breakdown and devulcanization selectivity; and (iii) the transfer of laboratory-optimized conditions to an industrial single-screw extrusion process, enabling assessment of changes in devulcanization efficiency and selectivity during scale-up.

## 2. Materials and Methods

### 2.1. Materials

Vulcanized EPDM rubber was supplied by SaarGummi (Madrid, Spain) and consisted of production non-conformities from door sealing profiles used in the automotive industry. The proprietary formulation could not be disclosed; however, its composition was characterized by thermogravimetric analysis yielding 22 wt.% EPDM, 26 wt.% carbon black (CB), 34 wt.% CaCO_3_, and 18 wt.% processing oil and low molecular mass additives. 2,2′-Dibenzamidodiphenyl disulfide (DBD, 98%) and hexadecylamine (HDA, >95%), were supplied by TCI Deutschland GmbH (via CymitQuimica, Barcelona, Spain) and used as devulcanizing agents. Cyclohexane (PA grade, Fisher Chemical, Waltham, MA, USA) was used as the swelling solvent for crosslink density determination. Acetone (analytical grade, Fisher Chemical) was employed for Soxhlet extraction. Both solvents were acquired from Enzymatic (Santo Antão do Tojal, Portugal). Paraffinic oil (7 phr) was added to 100 phr EPDM rubber as a processing aid in all devulcanization experiments.

### 2.2. Rubber Micronization

Prior to devulcanization, EPDM waste was granulated using a knife mill to 5–10 mm granules, following micronization into a fine powder < 1 mm, at room temperature. This procedure allows a better incorporation of the components for the following devulcanization process, increasing the specific surface area of rubber particles and promoting homogeneous heat and shear transfer during devulcanization.

### 2.3. Devulcanization Process

#### 2.3.1. Laboratory-Scale Devulcanization

Devulcanization experiments were performed in a Haake Polylab Rheomix 600 OS batch internal mixer (Thermo Scientific, Karlsruhe, Germany) equipped with two counter-rotating rotors and a total chamber volume of 69 cm^3^. Before mixing, the micronized EPDM was manually blended with the devulcanizing agent and paraffinic oil to achieve a homogeneous pre-mixture. A fixed charge mass of 61.17 g of micronized EPDM was used for all batches. Mixing was carried out for 20 min under the tested conditions, during which shear-induced devulcanization occurred.

Four temperature levels (160, 200, 220, and 280 °C) and two rotor speeds (100 and 150 rpm) were investigated. DBD was tested at 160 and 200 °C, while HDA was used at 220 and 280 °C, based on literature recommendations for the respective temperature ranges required for each agent [24,25,28,29]. Two agent concentrations (1 and 3 phr) were evaluated for each condition, in addition to blank experiments (0 phr agent) performed as controls. The full design of experiments, comprising 24 formulations, is presented in Table 1. Taking as an example the first Sample ID DBD-0-160-100, it can be read as “DBD system” with 0 phr DBD, at 160 °C and 100 rpm. Note each system was tested for 100 rpm and 150 rpm.

#### 2.3.2. Industrial Scale-Up Devulcanization

Industrial trials were performed in a continuous devulcanization line using an industrial single-screw extruder equipped with nine controlled heating zones. Each trial used 50 kg of EPDM waste and 3.5 kg of paraffinic oil, and it was pre-mixed using a Banbury mixer for 5 min before extrusion. The design of experiments is displayed in Table 2.

Based on the laboratory results, two processing temperatures were selected for scale-up: 200 °C for DBD and 280 °C for HDA. In each case, the temperature setpoint profile of each zone of the industrial extruder was the same along the nine controlled heating zones. The feed rate was 10 Hz and the screw speed in the devulcanization line was 19 Hz. A water-cooling section downstream of the hot zone was used to stop the devulcanization reaction. However, differences in equipment configuration, together with the lack of detailed information on the industrial extrusion process, prevented a direct comparison of the thermomechanical histories experienced by the material at laboratory and industrial scales. In particular, the absence of torque measurements from the industrial extrusion line excluded a quantitative comparison of mechanical energy input. Furthermore, local shear conditions, residence-time distributions, and actual material temperature profiles could not be assessed on an equivalent basis. Therefore, although similar processing temperatures were applied, these conditions should not be interpreted as representing equivalent thermomechanical histories across the two processing scales.

### 2.4. Material Characterization

#### 2.4.1. Crosslink Density and Degree of Devulcanization

The crosslink density was determined according to ASTM D6814-02 [30]. Approximately 10 g of each sample was first Soxhlet-extracted with acetone for 16 h to remove low-molecular-weight compounds. The acetone extraction step was performed prior to the swelling and soluble fraction calculations to remove processing oil and low-molecular-weight extractable additives. After extraction and drying (at 70 °C for 16 h), the resulting dry mass was taken as the reference mass for the subsequent calculations. The extracted samples were then placed in cyclohexane for 72 h at room temperature, and the solvent was replaced every 24 h to maintain swelling equilibrium. All swelling measurements were performed in triplicate (*n* = 3), which corresponds to 3 repeated analytical swelling measurements performed of the material obtained from a single devulcanization experiment for each processing condition. Therefore, the reported standard deviations demonstrate the reproducibility of the analytical procedure only. The reported values represent the average of the three measurements, and the standard deviation was below 3% of the mean for all samples.

The apparent crosslink density (Equation (1)) was calculated using the Flory–Rehner equation.

(1) $$\nu_{e}=\frac{-[\ln(1-V_{r})+V_{r}+\chi_{1}V_{r}^{2}]}{\left[V_{1}(V_{r}^{1/3}-V_{r}/2)\right]}$$

where ν_e_ is the apparent crosslink density (mol cm^−3^), V_1_ the solvent molar volume (108.7 cm^3^ mol^−1^ for cyclohexane), χ_1_ is the polymer–solvent interaction parameter (0.321 for EPDM-cyclohexane) [31], V_r_ is the volume fraction of rubber in the swollen sample given by Equation (2),

(2) $$V_{r}=\frac{m_{r}/\rho_{r}}{m_{r}/\rho_{r}+m_{s}/\rho_{s}}$$

where *m_r_* is the dry rubber mass and *ρ_r_* the dry rubber density (determined using Archimedes’ principle), *m_s_* is the mass of solvent absorbed by the polymer, and *ρ_s_* is the density of cyclohexane.

Because the compound was highly filled, Kraus correction was applied to obtain the corrected crosslink density (ν_e_corrected_) [26], following Equations (3) and (4).

(3) $$\nu_{e\_\mathrm{corrected}}=\frac{\nu_{e}}{1+K\times \Phi }$$

(4) $$\Phi =\frac{\varphi_{r}\times \rho_{r}\times m_{r0}}{\rho_{f}\times m_{r1}}$$

The Kraus correction was applied exclusively to the carbon black fraction, because this empirical parameter is specific to reinforcing carbon black and was not assumed to be applicable to CaCO_3_. Thus, *K* is the empirical parameter for CB (1.17 [32]), Φ is the volumetric fraction of CB, φ_r_ is the mass fraction of CB, *m_r_*_0_ is the dry mass before swelling in cyclohexane, *m_r_*_1_ is the dry mass after swelling in cyclohexane, and *ρ_f_* the density of the CB (g cm^−3^).

The devulcanization degree (%D) was calculated from the relative reduction in corrected crosslink density between the vulcanized reference and the devulcanized sample, Equation (5).

(5) $$\%D=\left(1-\frac{\nu_{e\_\mathrm{corrected}\;\mathrm{of}\;\mathrm{devulcanized}\;\mathrm{rubber}}}{\nu_{e\_\mathrm{corrected}\;\mathrm{of}\;\mathrm{reference}\;\mathrm{rubber}}}\right)\times 100\%$$

#### 2.4.2. Horikx Analysis

The main devulcanization mechanism was evaluated using Horikx theory, which relates the decrease in rubber crosslink density to the increase in soluble fraction generated during devulcanization. This method allows differentiation between two limiting mechanisms: random main-chain scission and selective crosslink scission [6,17,19]. It is particularly useful for sulfur-vulcanized EPDM, where both mechanisms may occur simultaneously during thermomechanical processing.

The soluble fraction calculation was based on the acetone-extracted and dried sample masses, after the removal of processing oil and low-molecular-weight compounds. The soluble fraction was determined according to Equation (6).

(6) $$\%s=\frac{m_{r0}-m_{r1}}{m_{r0}}\times 100\%$$

The Horikx curves were constructed following the methodology described by Seghar et al. [19]. If random main-chain scission is the dominant mechanism, the relative decrease in the effective number of chains in the gel fraction is described by Equation (7). If selective crosslink scission prevails, the correlation is given by Equation (8).

(7) $$1-\frac{\nu_{\mathrm{ef}}}{\nu_{\mathrm{ei}}}=1-\frac{{\left(1-\sqrt{s_{f}}\right)}^{2}}{{\left(1-\sqrt{s_{i}}\right)}^{2}}$$

(8) $$1-\frac{\nu_{\mathrm{ef}}}{\nu_{\mathrm{ei}}}=1-\frac{{\gamma_{f}\left(1-\sqrt{s_{f}}\right)}^{2}}{\gamma_{i}{\left(1-\sqrt{s_{i}}\right)}^{2}}$$

where *ν_ei_* and *ν_ef_* are the crosslink density, *s_i_* and *s_f_* the soluble fraction, and *γ_i_* and *γ_f_* the crosslinking indexes, before and after devulcanization. The latter can be calculated using Equation (9).

(9) $$s=\frac{\left(2+\gamma \right)-\sqrt{\gamma^{2}+4\gamma }}{2\gamma }$$

By plotting the experimental values relative to the two theoretical Horikx boundary curves (Equations (7) and (8)), it is possible to identify the dominant mechanism. Data points located closer to the random scission curve indicate significant polymer backbone degradation, whereas points approaching the selective crosslink scission curve indicate preferential cleavage of sulfur crosslinks with better preservation of the polymer chains. Points located between both curves suggest a mixed reclamation mechanism.

#### 2.4.3. Structural Analysis

Attenuated Total Reflectance-Fourier Transform Infrared (ATR-FTIR) spectroscopy was used as a complementary qualitative technique to monitor changes in the 800–700 cm^−1^ spectral region, including the weak band near 752 cm^−1^ usually associated with C–S containing structures (characteristic of sulfur crosslinks) [33]. Infrared spectra were recorded on a Jasco FT/IR-4X spectrometer in ATR mode coupled with a Germanium crystal, over a range of 4000–600 cm^−1^, at 4 cm^−1^ resolution and 32 accumulation (64 scans min^−1^).

#### 2.4.4. Thermogravimetric Analysis (TGA)

TGA of the vulcanized EPDM rubber was performed on a TA Instruments Q500 (New Castle, DE, USA), over a range of 40 to 800 °C, at a heating rate of 10 °C min^−1^, under nitrogen atmosphere. It was used to quantify the mass fractions of EPDM, processing additives, carbon black, and calcium carbonate in waste rubber.

#### 2.4.5. Rheological Characterization

Rheological behavior was analyzed in a moving die rheometer MDR 3000 Basic, from MonTech (MonTech Werkstoffprüfmaschinen GmbH, Buchen, Germany) at 200 °C, 0.833 Hz, and 0.2° amplitude for 3 min.

## 3. Results and Discussion

### 3.1. Vulcanized Waste EPDM Rubber Characterization

The material was a sulfur-cured EPDM rubber used in automotive sealing applications. Its high resistance to weathering, ozone, and thermal cycling is desirable in service, but these same attributes complicate circular reprocessing.

**Figure 1:**
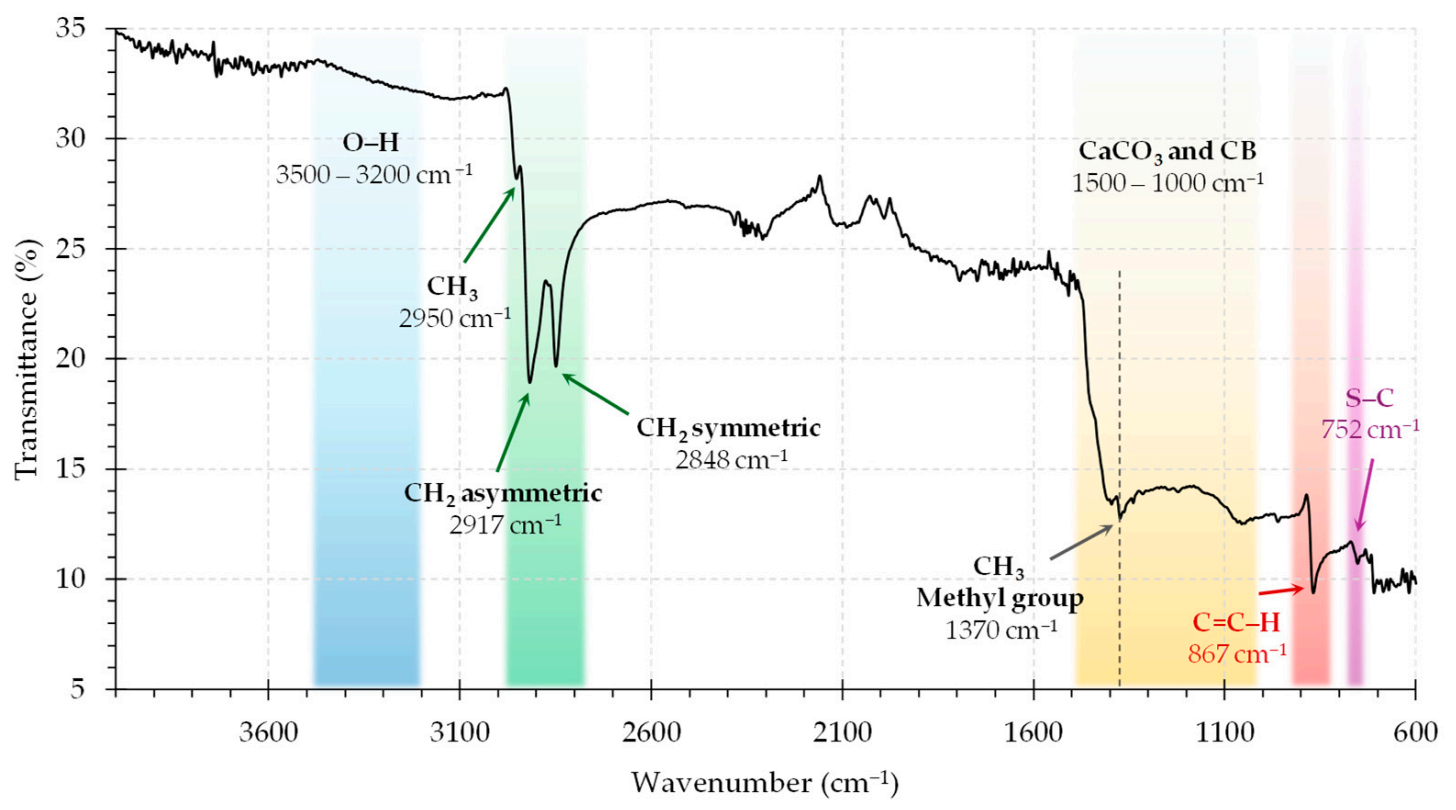
ATR-FTIR spectrum of vulcanized EPDM waste.

ATR-FTIR analysis (Figure 1) of the vulcanized rubber confirmed the EPDM nature of the waste, with the characteristic asymmetric and symmetric C–H stretching bands of CH_3_/CH_2_ groups at 2950, 2917 and 2848 cm^−1^ [33], the methyl vibration of the propylene units at 1370 cm^−1^ [34] and a broad shoulder between 3500 and 3200 cm^−1^ assigned to hydroxyl groups generated by mild oxidation during cure [35]. The S–C stretching mode at ~752 cm^−1^, associated with sulfidic crosslinks [34], was also detected. The strong absorption between 1500 and 1000 cm^−1^ is consistent with a high content of inorganic fillers, such as carbon black (CB) and calcium carbonate (CaCO_3_), and partly masks the absorption bands of the polymer backbone [35,36].

**Figure 2:**
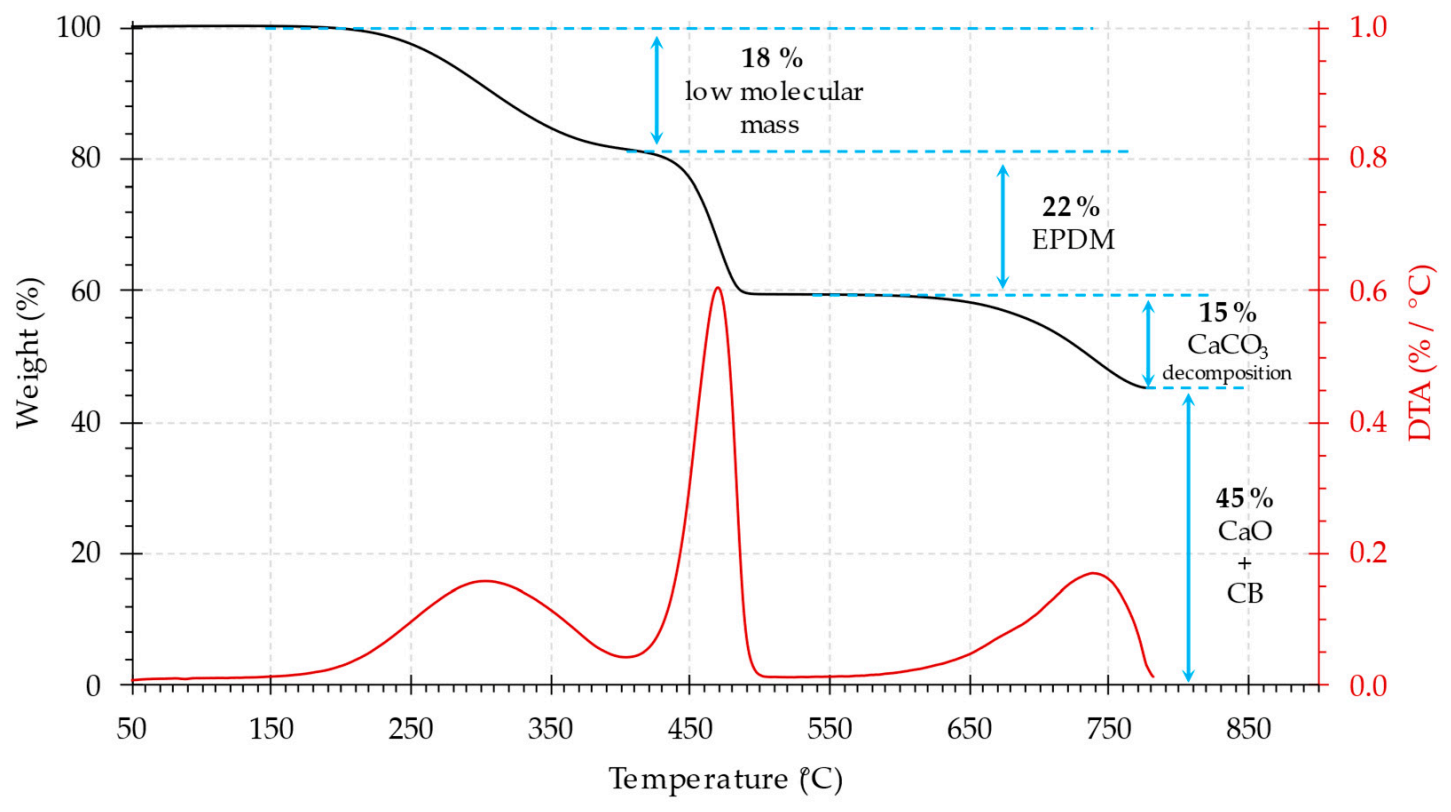
TGA result of waste EPDM rubber (**left**) and derivative thermogravimetry (**right**).

The thermogravimetric profile of the vulcanized EPDM waste (Figure 2) revealed a characteristic three-stage decomposition pattern. An initial mass loss of approximately 18% between 200 and 400 °C is attributed to the volatilization of low-molecular-weight components such as processing oils and organic additives [37]. A second stage between 400 and 500 °C, accounting for approximately 22% mass loss, corresponds to complete thermal degradation of the EPDM polymer chains [37]. A third stage between 500 and 800 °C is attributed to the conversion of CaCO_3_ to CaO, releasing carbon dioxide, typically with a yield of 47% [38]. The residual mass at 800 °C, considering that CB is thermally stable under inert atmosphere and that CaCO_3_ undergoes 47% mass loss with a 56% inorganic residue [39,40], allows the estimation of the CB and CaCO_3_ fractions. The analysis indicated that the compound contains approximately 22% EPDM polymer and 78% inorganic and organic additives, of which CB accounts for ~26% and CaCO_3_ for ~34% by mass. This exceptionally high filler content is consistent with the ATR-FTIR observations and underscores the critical importance of Kraus correction in all crosslink density calculations, as the filler phase severely restricts swelling equilibrium.

### 3.2. Study of Devulcanization on Laboratory Scale

#### 3.2.1. Devulcanization Efficiency on Laboratory Scale

Table 3 and Table 4 summarize the Kraus-corrected crosslink density (*ν*_corr_), degree of devulcanization (%D), and soluble fraction (%s) for all laboratory-scale experiments carried out at 100 and 150 rpm, respectively. The vulcanized EPDM reference showed a crosslink density of approximately 13.60 × 10^−5^ mol cm^−3^ and 2.5%s, which was used as the baseline for all devulcanization degree calculations.

In all experiments, the corrected crosslink density decreased relative to the vulcanized reference, confirming that thermomechanical treatment promoted partial devulcanization under every tested condition. Temperature was the most influential processing parameter. In general, increasing temperature reduced *ν*_corr_ and increased the degree of devulcanization, in agreement with previous studies [5,24]. Higher temperatures promote sulfur bond cleavage by increasing chain mobility and facilitating the action of the devulcanizing agents.

The effect of rotor speed was less pronounced within the tested range of 100–150 rpm. Only small differences were observed between both speeds, suggesting that this variation in shear rate was not sufficient to produce major changes in devulcanization efficiency. This indicates that, under the present conditions, temperature had a stronger influence than mechanical shear.

For the control experiments without devulcanizing agent (0 phr), substantial devulcanization degrees were observed, demonstrating that thermomechanical processing causes considerable network breakdown. The highest devulcanization degrees were observed at intermediate temperatures (200–220 °C) and the lower values were obtained at 160 °C, while only a limited improvement was observed at 280 °C. This behavior suggests the existence of an optimal temperature window for thermomechanical devulcanization. At low temperatures, the thermal energy is insufficient to promote effective network breakdown, whereas excessively high temperatures may favor undesirable secondary reactions, including main-chain scission and partial re-crosslinking, reducing process selectivity [41]. However, the reduction in crosslink density should not be evaluated independently of the selectivity of the scission process. As it will be demonstrated by the Horikx analysis, the 0 phr controls generally exhibit less selective reclamation behavior, characterized by a greater contribution from random main-chain scission. In contrast, the addition of DBD or HDA promotes preferential cleavage of sulfur crosslinks under the optimized conditions. Accordingly, the role of these chemical agents extends beyond increasing the apparent degree of devulcanization, as they also enhance the selectivity of network cleavage toward sulfur crosslinks.

For the DBD system, the best results were consistently obtained at 1 phr. At 160 °C, 1 phr DBD produced devulcanization degrees of 75.0 and 74.8% at 100 and 150 rpm, respectively, while 3 phr resulted in lower values of 66.6 and 65.3%. A similar trend was observed at 200 °C. The highest devulcanization degree of the entire study was obtained for DBD-1-200-100, reaching 85.6%. Increasing the DBD concentration to 3 phr reduced efficiency to approximately 70%.

This counterintuitive behavior at higher DBD concentration has been reported previously and is commonly associated with excessive formation of reactive sulfur radicals [23,24]. One possible explanation proposed in the literature is that when DBD is used above its optimal concentration, radical recombination may occur, promoting partial reorganization or re-crosslinking of the polymer network instead of net crosslink cleavage. These results indicate that DBD requires a controlled concentration to maximize selective devulcanization. However, these reactions were not directly characterized in the present study. Therefore, radical recombination and partial re-crosslinking should be regarded as plausible mechanistic interpretations of the observed concentration dependence rather than as experimentally demonstrated mechanisms.

For the HDA system, increasing the concentration from 0 to 3 phr generally reduced *ν*_corr_ and increased the degree of devulcanization at both temperatures studied. This behavior reflects the nucleophilic mechanism of HDA, in which the primary amine groups selectively attack sulfur crosslinks, particularly S–S and C–S bonds [20]. Unlike DBD, no reduction in efficiency was observed at higher HDA loading.

At 280 °C and 3 phr HDA (HDA-3-280-100), a devulcanization degree of 84.5% was obtained, which is comparable to the best DBD result. This confirms that HDA can also be highly effective, although it requires higher temperature and concentration to reach similar performance. The lower soluble fraction observed for this condition may indicate that crosslink cleavage occurred with limited backbone degradation, although this interpretation requires confirmation through Horikx analysis.

Overall, the results show that DBD and HDA follow different concentration–response relationships. DBD exhibits an optimum at low concentration, while HDA shows progressive improvement with increasing loading. Among all tested conditions, 1 phr DBD at 200 °C provided the best balance between high devulcanization efficiency and process selectivity, making it the most promising condition for further scale-up studies.

Another aspect worth considering is the potential influence of the high carbon black (CB) content of the EPDM waste (approximately 26 wt.%) on devulcanization efficiency, particularly through possible interactions with the devulcanizing agents. Preferential adsorption of DBD and HDA onto the CB surface, or other interfacial interactions, could potentially reduce the effective concentration of devulcanizing agents available to interact with sulfur crosslinks within the rubber network, thereby affecting the devulcanization process. However, this mechanism remains speculative, as no direct experimental evidence is currently available to support its contribution to the differences observed between the investigated systems. Further studies are therefore needed to clarify the role of high filler loadings and establish whether such interactions significantly influence the degree of devulcanization.

Additional information on material processability and thermomechanical behavior during laboratory-scale trials is provided in the Supplementary Materials (Figures S1–S3). Although a reduction in crosslink density was observed for the 0 phr formulation, the recovered EPDM remained in powder form, indicating that partial breakdown of the crosslinked network alone was insufficient to obtain a cohesive and processable rubber material. In contrast, the incorporation of devulcanizing agents promoted polymer chain mobility and rubber plasticization, facilitating material flow under thermomechanical processing conditions. These findings were consistent with the observed increases in torque, particularly for the formulations containing 3 phr DBD at 200 °C and 3 phr HDA at 280 °C. Nevertheless, as discussed in the Supplementary Materials, torque variations should be interpreted qualitatively rather than as direct indicators of devulcanization efficiency or selectivity, which were evaluated through swelling measurements and Horikx analysis.

#### 3.2.2. Devulcanization Selectivity on Laboratory Scale

**Figure 3:**
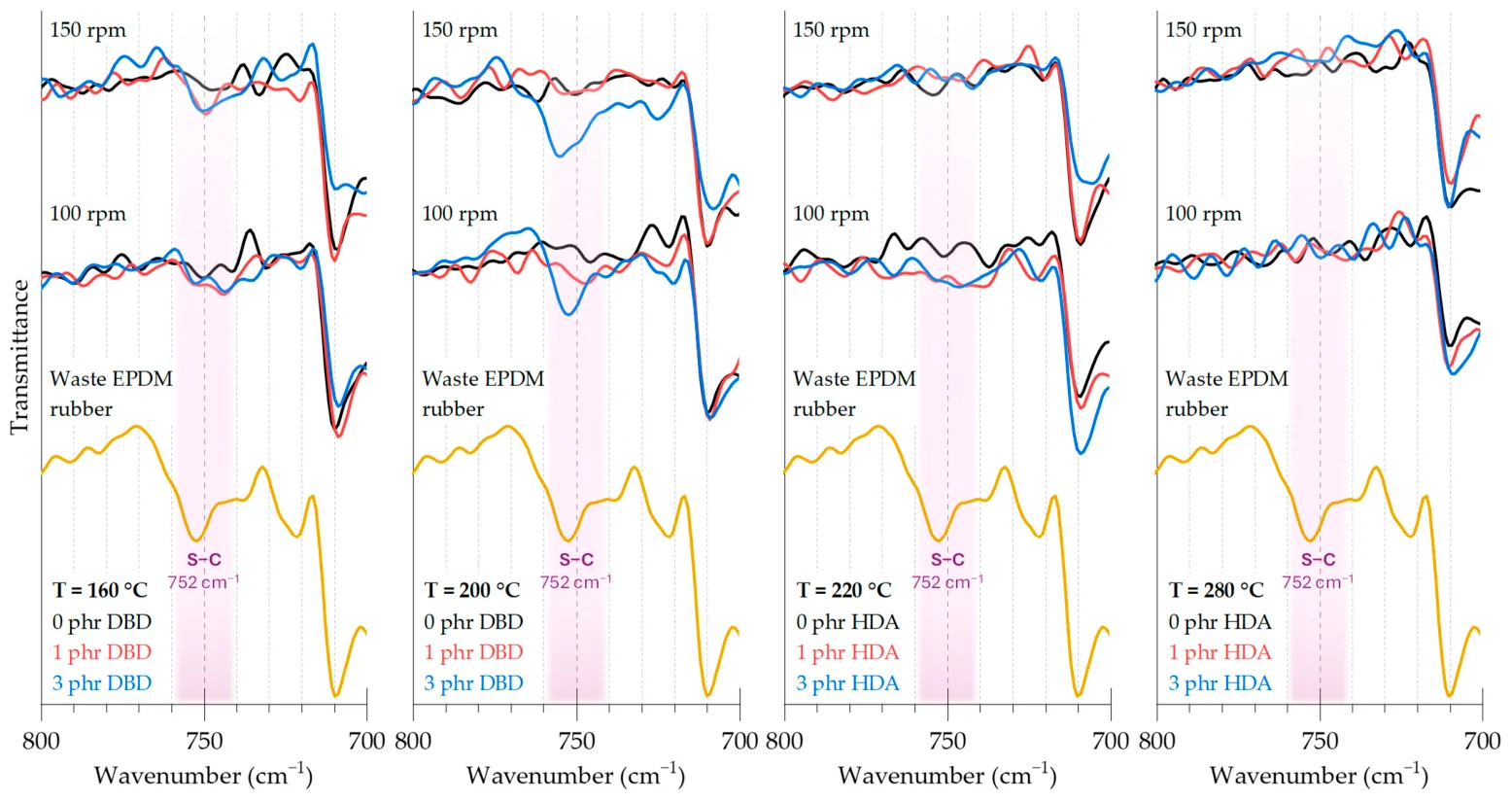
ATR-FTIR spectra of devulcanized systems on laboratory scale.

ATR-FTIR spectroscopy was used as a complementary technique to evaluate the structural changes induced by devulcanization, particularly in the 800–700 cm^−1^ region, associated with sulfur-containing bonds. Figure 3 shows the spectra of the devulcanized samples together with the vulcanized EPDM reference.

The vulcanized reference exhibited a visible absorption band at approximately 752 cm^−1^, commonly assigned to C–S stretching vibrations associated with sulfur crosslinks [20]. After devulcanization, this band showed progressive attenuation and, in some cases, became significantly less pronounced. This behavior suggests partial cleavage of sulfur-containing crosslinks during thermomechanical processing.

For the DBD-treated samples, the reduction in the C–S band was detectable but remained moderate, indicating partial disruption of the vulcanized network. In contrast, the HDA-treated samples showed a more pronounced decrease in this region, particularly at higher temperature and concentration. This trend is consistent with the swelling results, where HDA produced high degrees of devulcanization under severe conditions.

These observations may reflect the different mechanisms of the two devulcanizing agents. DBD acts mainly through a radical pathway, while HDA promotes nucleophilic attack on sulfur bonds, which may enhance selective cleavage of sulfide crosslinks [19]. However, it should be emphasized that ATR-FTIR is used here only as complementary qualitative evidence. The band near 752 cm^−1^ is relatively weak and might be affected by baseline variations, particle heterogeneity, and the high contents of CB and CaCO_3_. Therefore, changes in this region cannot provide direct or quantitative evidence of selective sulfur-crosslink cleavage, nor can they independently distinguish crosslink scission from polymer-backbone degradation. Therefore, ATR-FTIR alone cannot confirm the dominant scission mechanism and must be considered together with swelling and Horikx analysis.

**Figure 4:**
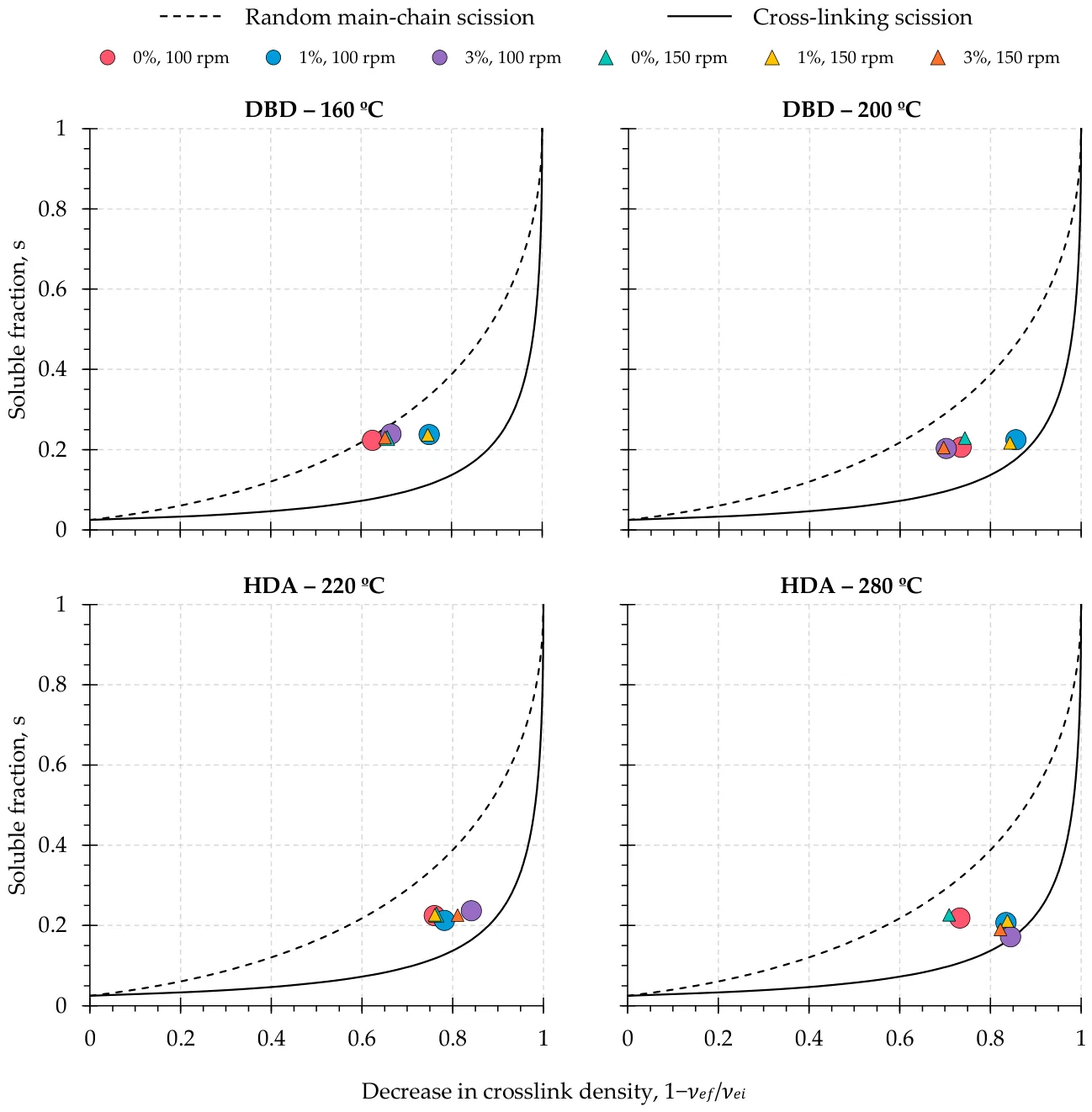
Horikx’s plot of laboratory-scale devulcanized samples: soluble fraction vs. the relative decrease in crosslink density.

Figure 4 presents the Horikx plots for all laboratory-scale devulcanization conditions. In most experiments, the soluble fraction remained within a relatively narrow range, typically between 19 and 24%, despite significant reductions in crosslink density. This behavior suggests that the polymer network underwent substantial crosslink cleavage without generating large amounts of extractable low-molecular-weight fragments. Such a trend is generally associated with preferential crosslink scission rather than extensive random chain degradation [14].

At 160 °C, most experimental points were located between the two theoretical Horikx boundary curves, indicating a mixed reclamation mechanism involving both crosslink rupture and main-chain scission. Under these conditions, the relatively low thermal activation of DBD likely limited its ability to selectively target sulfur crosslinks, resulting in less efficient and less selective devulcanization [23]. The addition of 1 phr DBD caused a slight shift of the data points toward the selective crosslink–scission curve, suggesting a modest improvement in selectivity.

At 200 °C, the DBD system showed a clearer improvement. The conditions DBD-1-200-100 and DBD-1-200-150 were positioned closer to the selective crosslink–scission curve, indicating that 1 phr DBD at this temperature provided the best balance between effective crosslink cleavage and preservation of the polymer backbone. This result is consistent with the role of DBD as a radical stabilizer that can favor sulfur bond cleavage when used within an appropriate concentration range [5]. Significantly, the corresponding 0 phr controls remain farther from the selective crosslink–scission boundary. Thus, despite their relatively high apparent devulcanization degrees, thermomechanical treatment alone produces less selective network breakdown, with a comparatively greater contribution from polymer main-chain scission. This distinction demonstrates that the effect of DBD should be evaluated not only from the magnitude of the crosslink-density reduction but also from the improved selectivity indicated by the Horikx analysis.

For the HDA system at 220 and 280 °C, most data points were also located closer to the selective crosslink–scission boundary than the DBD experiments performed at 160 °C. This suggests improved selectivity under these conditions and supports the effectiveness of the nucleophilic mechanism of HDA. At 280 °C, increasing HDA concentration shifted the points further toward the selective devulcanization region, indicating that higher HDA loading promoted more efficient sulfur bond cleavage.

Nevertheless, none of the experimental points reached the theoretical selective crosslink–scission boundary. This is likely related to the efficient vulcanization system of the original EPDM compound, which probably contains a significant fraction of monosulfidic crosslinks. These bonds are more thermally stable and more difficult to selectively cleave than polysulfidic linkages, increasing the likelihood of simultaneous backbone degradation during processing [20].

Overall, the Horikx analysis indicates that selective devulcanization was improved under optimized conditions, particularly for DBD at 200 °C and HDA at 280 °C. However, complete selectivity was not achieved, and all systems showed some degree of mixed reclamation behavior. This highlights the difficulty of preserving polymer integrity during thermomechanical devulcanization of highly filled EPDM compounds.

Although the combined swelling, Horikx, ATR-FTIR, and rheological results are consistent with preferential sulfur-crosslink cleavage under the optimized conditions, further molecular weight distribution analysis (GPC) of the EPDM chains should be performed. Therefore, the preservation of the polymer backbone cannot be convincingly demonstrated from the present dataset, and these results should be taken as evidence of preferential crosslink scission within an overall mixed reclamation mechanism.

### 3.3. Study of Devulcanization at Industrial Scale

Table 5 summarizes the results obtained in the industrial single-screw devulcanization line. Compared with the laboratory-scale internal mixer, the industrial process showed a clear reduction in devulcanization efficiency for both systems.

For the reference experiments without devulcanizing agent, the degree of devulcanization reached only 25.9% at 200 °C and 32.0% at 280 °C. These values are substantially lower than those obtained in the laboratory under similar temperatures, where devulcanization degrees above 70% were achieved. This confirms that the industrial extrusion process was significantly less effective in promoting network breakdown under the tested conditions.

The addition of 1 phr DBD at 200 °C increased the degree of devulcanization from 25.9 to 59.6% and reduced the corrected crosslink density from 10.0 to 5.45 × 10^−5^ mol cm^−3^. This represents a substantial improvement and confirms that DBD remains effective even under the less favorable processing conditions of the continuous extrusion line. However, the result is still considerably lower than the 85.6% achieved at laboratory scale for the same agent concentration.

In contrast, the addition of 1 phr HDA at 280 °C did not improve the process. The degree of devulcanization slightly decreased from 32.0 to 26.9%, suggesting that HDA was not effective under the industrial conditions used in this study. This behavior indicates that the nucleophilic mechanism of HDA may be more sensitive to processing limitations such as mixing efficiency and residence time.

Although differences in mixing efficiency, residence time, shear history, and thermomechanical conditions could explain the lower industrial devulcanization degrees, these interpretations remain hypothetical and require experimental validation. Therefore, these results should be regarded as a preliminary evaluation of the optimized laboratory conditions to the industrial scale-up, rather than evidence of the individual effects of specific processing parameters.

First, the laboratory-scale process was performed in an intensive internal mixer equipped with counter-rotating rotors, which provides high local shear stress and efficient distributive and dispersive mixing. In contrast, the industrial process used a conventional single-screw extruder, where material transport occurs mainly by drag flow along the barrel wall. This configuration provides limited back-mixing and lower local shear intensity. As a result, the devulcanizing agent may not be distributed homogeneously throughout the highly filled rubber matrix, reducing its effective utilization.

Second, residence time in the industrial extruder was considerably shorter than the 20 min used in the laboratory mixer and could not be independently controlled. Since thermomechanical devulcanization is a kinetically controlled process, shorter residence times directly limit the extent of sulfur bond cleavage. This effect is expected to be particularly important for HDA, whose nucleophilic mechanism requires sufficient contact time between the amine and sulfur crosslinks. The poor performance of HDA at industrial scale suggests that the reaction time may have been insufficient for effective devulcanization.

Third, the screw geometry did not include dedicated mixing elements, and the local shear history could not be matched to the laboratory conditions. Lower peak shear stresses reduce the mechanochemical contribution to bond scission, which is an important part of thermomechanical devulcanization [8,28]. The combined effect of lower shear, reduced mixing, and shorter residence time explains the significant drop in efficiency observed during scale-up. Because shear rate, shear stress, and residence-time distribution were not directly quantified, the lower industrial devulcanization efficiency cannot be attributed to any single processing parameter. The observed differences may therefore be associated with the combined effects of less mixing intensive, a different shear history, and shorter residence time distribution in the single-screw extruder.

**Figure 5:**
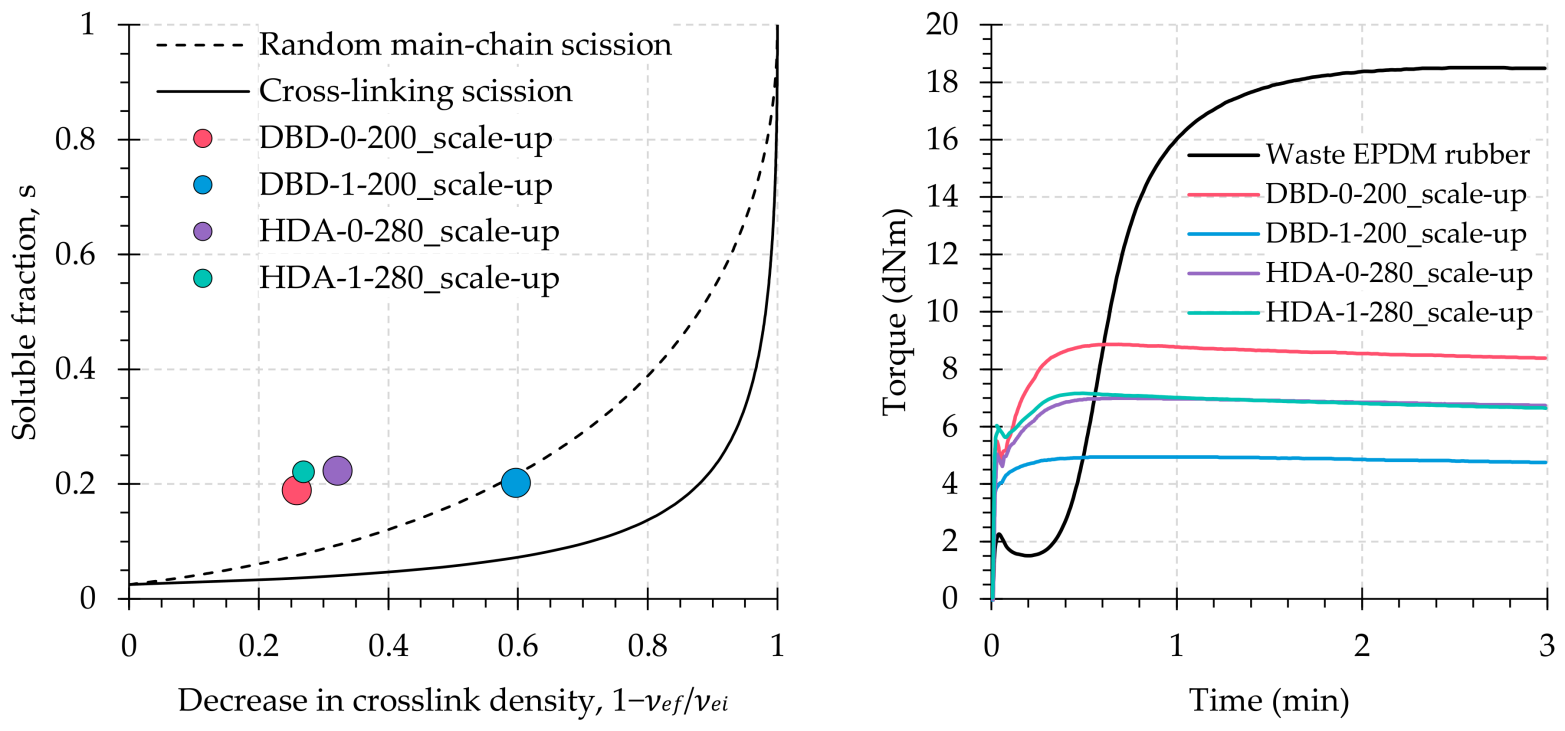
Horikx’s plot of Industrial scale-up devulcanized samples: soluble fraction vs. the relative decrease in crosslink density (**left**), and MDR rheometry (**right**).

Horikx analysis (Figure 5) provides additional insight into the mechanism changes observed at industrial scale. In the laboratory-scale optimized conditions, such as DBD-1-200-100 and HDA-3-280-100, the experimental points were located closer to the selective crosslink–scission boundary, indicating relatively good selectivity. In contrast, the industrial-scale samples were displaced upward and shifted toward the random main-chain scission region, where several experimental points showed a soluble fraction higher than would be expected according to the theoretical Horikx curves.

It should be noted that the Horikx model assumes homogeneous network breakdown throughout the vulcanized material, with no macroscopic spatial gradients across the rubber particles. Accordingly, the increase in soluble fraction is expected to remain within the bounds defined by the two theoretical Horikx curves. However, the experimental data obtained from the industrial-scale trials show that larger reductions in crosslink density are accompanied by relatively high soluble fractions, suggesting a less selective network-cleavage process. One possible explanation is the so-called “peeling mechanism” proposed by Dierkes et al. [42,43], in which devulcanization occurs mainly at the outer surface of the rubber particles. In this case, the particle shell undergoes extensive degradation and generates a significant soluble fraction, while the core remains largely vulcanized. As a result, swelling measurements reflect an average crosslink density of a heterogeneous particle, while the extracted soluble fraction is dominated by the degraded surface region.

Although this mechanism was not directly confirmed, it is consistent with the limited mixing and short residence time of the extrusion process. The existence of radial temperature and composition gradients inside the particles is therefore a plausible explanation for the reduced selectivity observed at industrial scale. Nevertheless, the Horikx analysis provides only indirect evidence for this interpretation. No spatially resolved measurement of network degradation was performed, and therefore the occurrence of a peeling mechanism cannot be directly confirmed in the present study. Further characterization would be required to establish whether a crosslink-density gradient exists from the particle surface toward the core.

Moving die rheometry (MDR) further supported the swelling and Horikx results. The DBD-1-200_scale-up sample showed the lowest torque throughout the test and the lowest final maximum torque (MH), indicating the greatest reduction in residual crosslink density and improved processability. In contrast, DBD-0-200_scale-up retained a higher torque profile, consistent with a more preserved network structure.

The two samples processed at 280 °C, HDA-0-280_scale-up and HDA-1-280_scale-up, showed very similar rheological behavior, confirming that HDA did not provide a measurable improvement under industrial conditions. Their higher MH values relative to DBD-1-200_scale-up indicate a higher residual crosslink density and more limited processing freedom for subsequent compounding.

Overall, the industrial trials demonstrate that laboratory-scale optimization cannot be directly transferred to continuous processing without considering equipment design and process kinetics. Among the tested conditions, DBD at 1 phr and 200 °C showed the best industrial performance and appears to be the most promising route for further process optimization. Future improvements should focus on screw configuration, residence time control, and enhanced mixing efficiency to increase both devulcanization degree and selectivity during industrial-scale processing.

### 3.4. Implications for Circular Economy and Polymer Sustainability

The results of this study have direct relevance for improving the circularity of vulcanized rubber waste, particularly in the automotive sector. Global rubber production exceeds 30 million tonnes per year [1], and European regulations require that at least 95% of end-of-life vehicles be recovered by weight [5]. These requirements increasingly limit disposal-based strategies for rubber components and reinforce the need for material recovery routes that preserve the value of the polymer.

The present work demonstrates that thermomechanical devulcanization can be applied successfully even to highly filled industrial EPDM waste streams. Devulcanization degrees above 85% were achieved at laboratory scale, while 60% was obtained under preliminary industrial-scale conditions using a single-screw extruder. This is particularly relevant because the studied waste contained only approximately 22 wt.% polymer and a very high fraction of inorganic fillers, mainly carbon black and calcium carbonate. Such compositions are common in real industrial waste streams but are often underrepresented in devulcanization studies, which frequently focus on simplified model formulations [44,45]. The results therefore provide practical evidence that selective network breakdown is still achievable in complex commercial compounds.

From a processing perspective, the MDR results suggest that the devulcanized materials retain the ability to undergo further processing under flow conditions. All industrial-scale samples showed measurable torque development and well-defined curing plateaus, although at lower levels than the original vulcanized material. In particular, the DBD-1-200_scale-up sample showed the largest reduction in residual crosslink density while maintaining a meaningful torque difference between minimum and maximum torque ΔM (MH − ML). This behavior is consistent with the presence of residual reactive sites that could, in principle, participate in subsequent crosslinking reactions. However, the actual revulcanization capability and the resulting mechanical properties were not assessed in the present study and remain to be demonstrated. These observations should therefore be regarded as preliminary indicators of processability rather than as evidence of practical recyclability, which would require full revulcanization cycles and mechanical characterization that are beyond the scope of the present work.

This distinction is important for circular economy applications. Mechanical grinding and conventional reclamation often produce materials that can only be reused as low-value fillers, with limited contribution to true material circularity [8,10]. In contrast, selective devulcanization aims to restore processability while preserving sufficient polymer integrity for re-incorporation into new rubber products. Although full revulcanization trials and mechanical property evaluation were not included in this study, the present results indicate that this approach is technically feasible and justify further investigation.

From an environmental perspective, thermomechanical devulcanization offers clear advantages over disposal routes such as incineration and landfill, as well as over high-temperature treatments such as pyrolysis. Processing temperatures of 200–280 °C are significantly lower than those typically required for thermal decomposition processes, reducing energy demand and preserving the polymer structure for reuse. In addition, the relatively low amount of devulcanizing agent required (1–3 phr) compares favorably with chemical devulcanization routes that rely on larger reagent quantities or solvent-intensive processes [28], Nevertheless, the safe handling and environmental management of agents such as DBD and HDA remain important considerations for industrial implementation.

The comparison between laboratory and industrial-scale results also highlights an important practical challenge: achieving high devulcanization efficiency is not sufficient if process selectivity is lost during scale-up. The reduction in performance observed in the single-screw extruder demonstrates that equipment design, residence time, and mixing efficiency strongly influence the final material quality. This reinforces the importance of combining crosslink density measurements with mechanistic tools such as Horikx analysis when evaluating industrial viability.

Finally, the framework established in this study—combining swelling analysis with Kraus correction, Horikx interpretation, ATR-FTIR support, and industrial comparison—provides a useful methodology for future work on sulfur-vulcanized elastomers. The different concentration-response behaviors observed for DBD and HDA also provide practical guidance for process optimization. DBD showed an optimum concentration window, while HDA required higher temperature and loading to achieve comparable results. These findings are relevant not only for EPDM recycling but also for the broader development of more selective and industrially viable recycling strategies for crosslinked polymer systems.

## 4. Conclusions

This study investigated the thermomechanical devulcanization of highly filled sulfur-vulcanized EPDM rubber waste from automotive production using DBD and HDA as devulcanizing agents under laboratory and preliminary industrial-scale conditions. The results demonstrated that both agents were capable of reducing the crosslink density of the vulcanized network, although they followed different concentration–response behaviors and exhibited distinct sensitivities to processing conditions.

At laboratory scale, DBD showed the best overall performance, reaching a devulcanization degree of approximately 86% at 1 phr and 200 °C. Increasing the DBD concentration to 3 phr reduced the devulcanization efficiency, suggesting that excessive radical formation may promote partial re-crosslinking or network reconstruction, as proposed in previous studies, although these mechanisms were not directly verified in the present work. In contrast, HDA exhibited progressive improvement with increasing concentration and achieved approximately 85% devulcanization at 3 phr and 280 °C, consistent with its nucleophilic mechanism for sulfur bond cleavage.

Horikx analysis indicated that optimized processing conditions promoted preferential crosslink scission with limited but unavoidable polymer backbone degradation. Among the investigated conditions, 1 phr DBD at 200 °C and higher HDA concentrations at 280 °C showed the highest selectivity toward sulfur crosslink cleavage. Nevertheless, none of the experimental conditions reached the theoretical selective-scission boundary, indicating that all systems still involved a mixed reclamation mechanism. The presence of highly stable monosulfidic crosslinks and the exceptionally high filler content of the industrial EPDM waste likely contributed to the limited selectivity observed. Since these results are indicative, future work should include molecular weight distribution analysis to infer the true state of the molecular structure of EPDM.

The industrial-scale trials performed in a single-screw extrusion process resulted in significantly lower devulcanization efficiency than laboratory-scale processing. The best industrial result was obtained using 1 phr DBD at 200 °C, reaching approximately 60% devulcanization. The differences between laboratory and industrial-scale performance might be related to distinct thermomechanical conditions, like mixing, residence-time, shear or thermal histories. However, as these parameters were not quantified on a comparable basis, their individual contributions to devulcanization efficiency and selectivity could not be reliably determined.

The combined swelling, Horikx, ATR-FTIR, and rheological analyses demonstrated that thermomechanical devulcanization can be successfully applied to highly filled industrial EPDM waste streams, even in compounds containing very high levels of inorganic fillers. The results also highlight the importance of combining crosslink-density analysis with mechanistic interpretation to properly evaluate process selectivity during scale-up.

The MDR results provide evidence of the processability of the devulcanized materials under the applied test conditions. However, these findings alone are insufficient to confirm the preservation of reactive sites capable of supporting subsequent revulcanization. Therefore, full revulcanization studies and mechanical-property evaluation are still required to confirm their practical reuse potential in new EPDM formulations. Additional studies involving morphology analysis, molecular-weight characterization, and optimization of industrial screw configuration and residence-time distribution would further improve the understanding and industrial applicability of the process.

Overall, DBD at 1 phr and 200 °C was identified as the most promising condition among those investigated. The study demonstrates that thermomechanical devulcanization is a viable route for the recovery of highly filled EPDM rubber waste while also emphasizing that process control, equipment design, and selectivity optimization remain critical challenges for successful industrial implementation and circular reintegration of devulcanized elastomers.

## Figures and Tables

**Table 1:** Design of experiments at laboratory scale devulcanization for 100 phr EPDM and 7 phr oil.

System	Sample ID	Agent (phr)	Temp (°C)	Speed (rpm)
DBD	DBD-0-160-100/150	0	160	100/150
	DBD-1-160-100/150	1		
	DBD-3-160-100/150	3		
	DBD-0-200-100/150	0	200	
	DBD-1-200-100/150	1		
	DBD-3-200-100/150	3		
HDA	HDA-0-220-100/150	0	220	
	HDA-1-220-100/150	1		
	HDA-3-220-100/150	3		
	HDA-0-280-100/150	0	280	
	HDA-1-280-100/150	1		
	HDA-3-280-100/150	3		

**Table 2:** Design of experiments at Industrial scale-up devulcanization for 100 phr EPDM and 7 phr oil.

System	Sample ID	Agent (phr)	Temp (°C)	Output (kg/h)
DBD	DBD-0-200_scale-up	0	200	100
	DBD-1-200_scale-up	1		
HDA	HDA-0-280_scale-up	0	280	
	HDA-1-280_scale-up	1		

**Table 3:** Laboratory-scale results for 100 rpm speed from swelling tests after Kraus correction.

System	Sample ID	ν_e_ (×10^−5^ mol cm^−3^) *	%D (%) *	%s (%) *
DBD	DBD-0-160-100	5.11	62.4	22.3
	DBD-1-160-100	3.40	75.0	23.7
	DBD-3-160-100	4.54	66.6	24.0
	DBD-0-200-100	3.60	73.5	20.6
	DBD-1-200-100	1.96	85.6	22.5
	DBD-3-200-100	4.05	70.2	20.3
HDA	HDA-0-220-100	3.28	75.9	22.5
	HDA-1-220-100	2.98	78.1	21.3
	HDA-3-220-100	2.16	84.1	23.7
	HDA-0-280-100	3.63	73.3	21.9
	HDA-1-280-100	2.26	83.4	20.8
	HDA-3-280-100	2.11	84.5	17.2

* Standard deviation is below 3% of the mean value (n = 3). These measurements are analytical replicates and not independent devulcanization replicates of the same system.

**Table 4:** Laboratory-scale results for 150 rpm speed from swelling tests after Kraus correction.

System	Sample ID	ν_e_ (×10^−5^ mol cm^−3^) *	%D (%) *	%s (%) *
DBD	DBD-0-160-150	4.66	65.7	22.9
	DBD-1-160-150	3.43	74.8	23.7
	DBD-3-160-150	4.72	65.3	23.0
	DBD-0-200-150	3.50	74.3	22.9
	DBD-1-200-150	2.14	84.3	21.6
	DBD-3-200-150	4.13	69.6	20.5
HDA	HDA-0-220-150	3.28	75.9	22.5
	HDA-1-220-150	3.25	76.1	22.7
	HDA-3-220-150	2.57	81.1	22.6
	HDA-0-280-150	3.96	70.9	22.6
	HDA-1-280-150	2.22	83.7	21.1
	HDA-3-280-150	2.42	82.2	19.1

* Standard deviation is below 3% of the mean value (n = 3). These measurements are analytical replicates and not independent devulcanization replicates of the same system.

**Table 5:** Industrial-scale results from swelling tests after Kraus correction.

System	Sample ID	ν_e_ (×10^−5^ mol cm^−3^) *	%D (%) *	%s (%) *
DBD	DBD-0-200_scale-up	10.08	25.9	18.9
	DBD-1-200_scale-up	5.49	59.6	20.3
HDA	HDA-0-280_scale-up	9.25	32.0	22.4
	HDA-1-280_scale-up	9.94	26.9	22.2

* Standard deviation is below 3% of the mean value (n = 3). These measurements are analytical replicates and not independent devulcanization replicates of the same system.

## Data Availability

The original contributions presented in this study are included in the article/Supplementary Material. Further inquiries can be directed to the corresponding author.

## Supplementary Materials

The following supporting information can be downloaded at: https://www.mdpi.com/article/10.3390/ma19214559/s1. Figure S1. (a) Size reduction of the EPDM waste: as-received (left), knife-milled granules (5–10 mm) (center), and micronized powder (<1 mm) (right). (b) Example of laboratory batch mixer devul-canization trials without and with 3 phr of DBD and HDA, at 200 °C and 280 °C, respectively, at 150 rpm for 20 min. Similar visual results were obtained for 1 phr systems. Figure S2. Evolution of torque and melt temperature over time, measured on the laboratory batch mixer devulcanization trials with DBD, at 160 °C and 200 °C, at 100 (a and c) and 150 (b and d) rpm, respectively. Figure S3. Evolution of torque and melt temperature over time, measured on the laboratory batch mixer devulcanization trials with HDA, at 220 °C and 280 °C, at 100 (a and c) and 150 (b and d) rpm, respectively.

## Abbreviations

The following abbreviations are used in this manuscript:

ATR-FTIR	Attenuated Total Reflectance-Fourier Transform Infrared Spectroscopy
CB	Carbon Black
DBD	2,2′-Dibenzamidodiphenyl Disulfide
EPDM	Ethylene–Propylene–Diene Monomer rubber
HDA	Hexadecylamine
MDR	Moving Die Rheometer
TGA	Thermogravimetric Analysis
MH	Maximum Torque
ML	Minimum Torque
phr	Parts per Hundred Rubber
rpm	Revolutions per Minute
