# Supplementary material for: Thermomechanical Devulcanization of Highly Filled EPDM Rubber Waste: Process Optimization, Selectivity Assessment, and Preliminary Industrial Scale-Up
Source: Materials (Basel). 2026 Oct 29;19(21):4559. doi: 10.3390/ma19214559 (PMC13764389; doi:10.3390/ma19214559)
Supplement: Supplementary file 1 [file materials-19-04559-s001.zip › materials-4311803-supplementary.pdf]

materials MDPI

Article – Supplementary material

Thermomechanical Devulcanization of Highly Filled EPDM Rubber Waste: Process Optimization, Selectivity Assessment, and Preliminary Industrial Scale-Up

Pedro Veiga Rodrigues, Mateus Vieira Ribeiro, Ana Vera Machado *

Department of Polymer Engineering, Institute for Polymers and Composites (IPC), University of Minho, 4804-533 Guimarães, Portugal; pedro.rodrigues@dep.uminho.pt (P.V.R.); mateusvieiraribeiro.23@gmail.com (M.V.R.)
* Correspondence: avm@dep.uminho.pt

Supplementary material

The supplementary material presented gives details about the laboratory-scale trials. Figure S1 a) shows the size reduction process of EPDM waste prior to the devulcanization trials. Figure S1 b) shows 4 examples of laboratory batch mixer trials after devulcanization with 0 and 3 phr of devulcanizing agent, comparing how the material looked like after devulcanization. Figures S2 and S3 show the evolution of mixer torque and melt temperature (temperature inside the mixer chamber) for 20 min, on the laboratory-scale devulcanization trials. This extra information gives details about the lab-scale devulcanization experiments reported in the main paper.

As displayed in Figure S1 a), the particle size of EPDM waste rubber was reduced up to a fine powder (<1mm) to facilitate the incorporation into the internal mixer. The processed material without the incorporation of devulcanization agent remained largely in powder form (Figure S1 b)), without any significant change in the mixer torque and temperature (Figure S2 and S3), suggesting that the thermomechanical devulcanization alone is not sufficient to produce a final cohesive rubber sample. On the other hand, the addition of devulcanization agents led to a significant different outcome. When 3 phr of devulcanizing agent was used, the recovered samples formed a cohesive and deformable material. Although these observations are only qualitative, they suggest that the use of devulcanization agents is essential to restore the processability of the vulcanized EPDM waste.

When looking at the torque and temperature profiles (Figures S2 and S3), the torque and temperature signals are influenced by several factors beyond the viscosity or crosslink density of the rubber. The degree of interaction between EPDM waste and internal mixing rotors affect the measured resistance for the rotors to rotate (torque) and viscous dissipation (temperature). For instance, when the rubber powder remains loose, the resultant torque is low because the material can freely flow under the rotation of the rotors. When the particles begin to agglomerate and form a cohesive mass, the rotor resistance increases and, therefore, the torque increases. Therefore, an increase in torque does not necessarily imply an increase in crosslink density, but may instead reflect the progressive plastication ability of the rubber under thermomechanical processing conditions, leading to the formation of a cohesive and processable rubber mass (as seen in Figure S1 b)).

Almost all experiments with 0 and 1 phr show a decreasing torque over time (Figure S2 and S3), expect for HDA at 280 ºC (Figure S3 c) and d)). As for the DBD systems, only 3 phr was able to increase the torque under mixing conditions, which may be related to the formation of a more cohesive rubber mass (as shown in Figure S1 b)). This behavior

should not the only attributed to network breakdown, as swelling results show that increasing DBD concentration did not improve the degree of devulcanization (possibly due to recombining effects). Therefore, an increase in processability does not necessarily coincide with a greater reduction in crosslink density. Secondary reactions may also contribute to the observed torque variation but torque itself is not enough to conclude on the devulcanization mechanisms involved.

HDA systems show a different behavior (Figure S3). At 220 ºC, torque decreases on all systems, with a minor increase in torque near the end of the devulcanization process for 3 phr at 150 rpm (Figure S3 b)), suggesting that plastication could occour for higher reaction times. At 280 ºC, torque profiles exhibit a distinct peak, followed by a decrease in torque intensity. One possible explanation is that the initial reaction between HDA and crosslinked EPDM enables the chain breakdown, increasing polymer chain mobility and facilitating rubber plastication, allowing the material to flow under thermomechanical processing conditions. Nevertheless, these observations do not provide solid evidence to identify the main mechanisms responsible for the torque variations.

Therefore, torque analysis should only be considered as qualitative analysis, rather than quantitation. These results show some relationship between crosslink density and material processability recovery. As an overall analysis, thermomechanical treatment alone (without devulcanizing agents) was able to reduce the crosslink density, yet keeping its powder form. On the other hand, the use of devulcanizing agent (DBD or HDA) was important to retrieve the processability ability of the EPDM matrix. However, neither torque or visual appearance of the recovered rubber can determine the extent or selectivity of EPDM devulcanization. For this reason, Horikx analysis is an essential tool to access these devulcanization mechanisms.

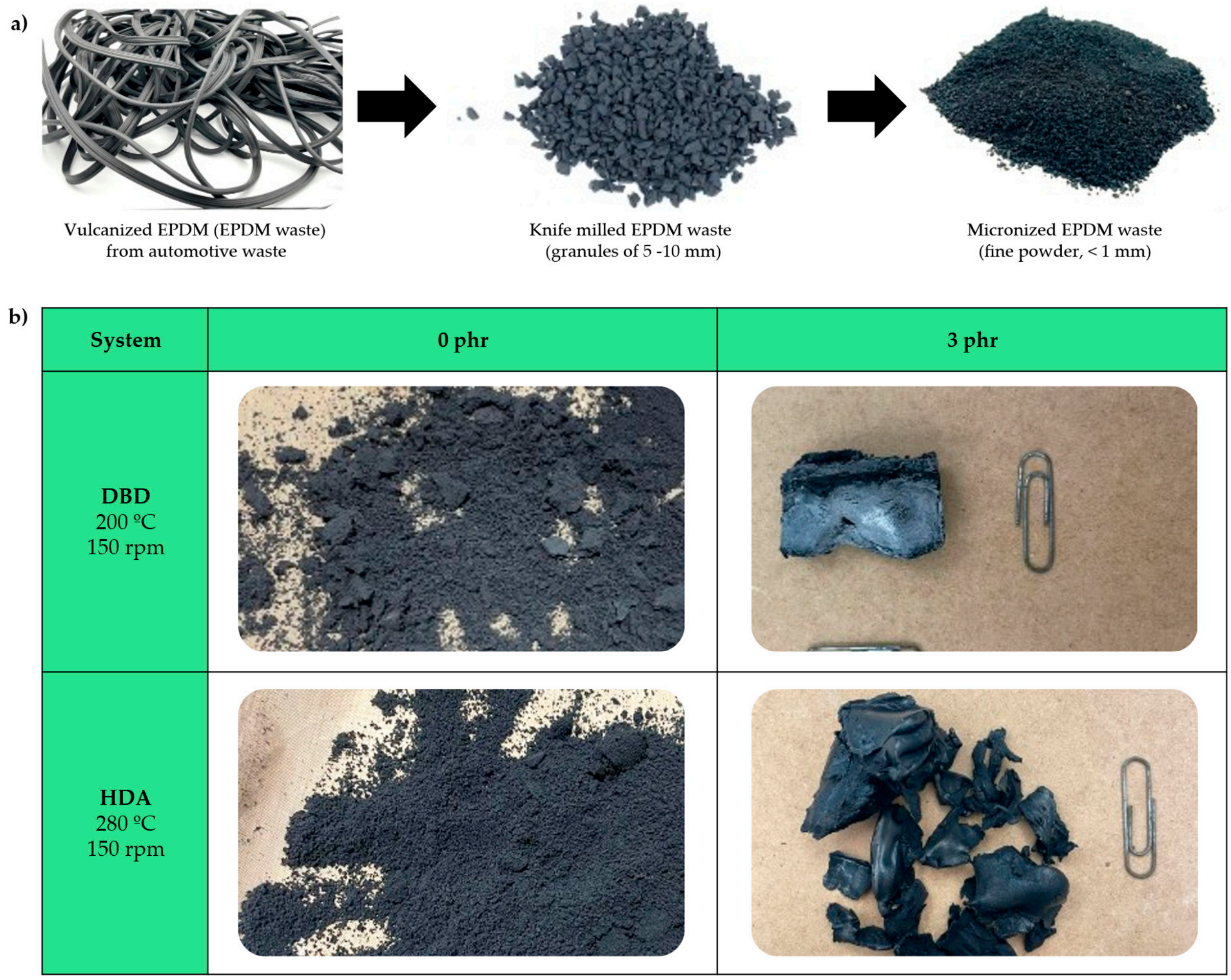

Figure S1. a) Size reduction of the EPDM waste: as-received (left), knife-milled granules (5–10 mm) (center), and micronized powder (<1 mm) (right). b) Example of laboratory batch mixer devulcanization trials without and with 3 phr of DBD and HDA, at 200 ºC and 280 ºC, respectively, at 150 rpm for 20 minutes. Similar visual results were obtained for 1 phr systems.

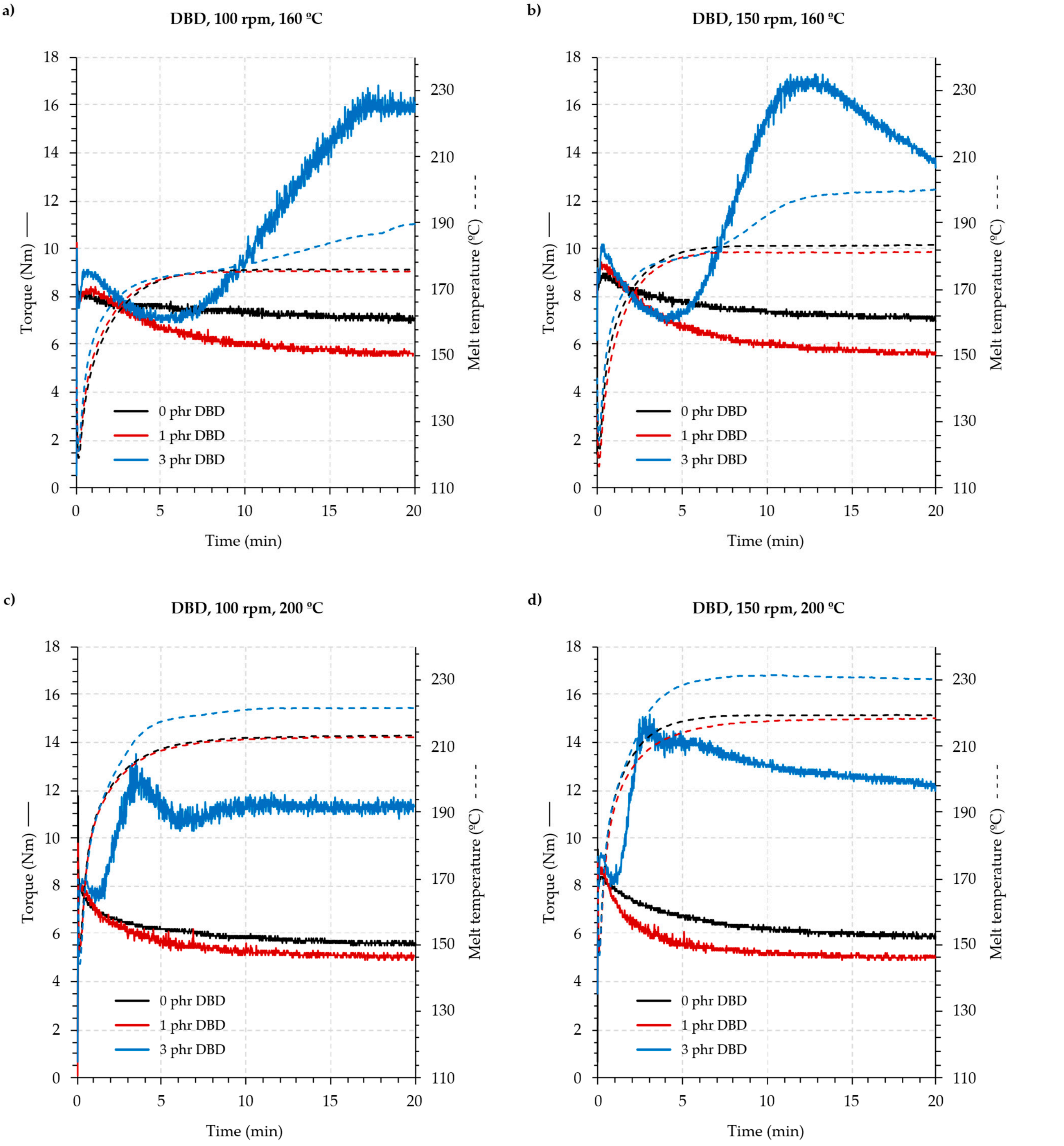

Figure S2. Evolution of torque and melt temperature over time, measured on the laboratory batch mixer devulcanization trials with DBD, at 160 ºC and 200 ºC, at 100 (a and c) and 150 (b and d) rpm, respectively.

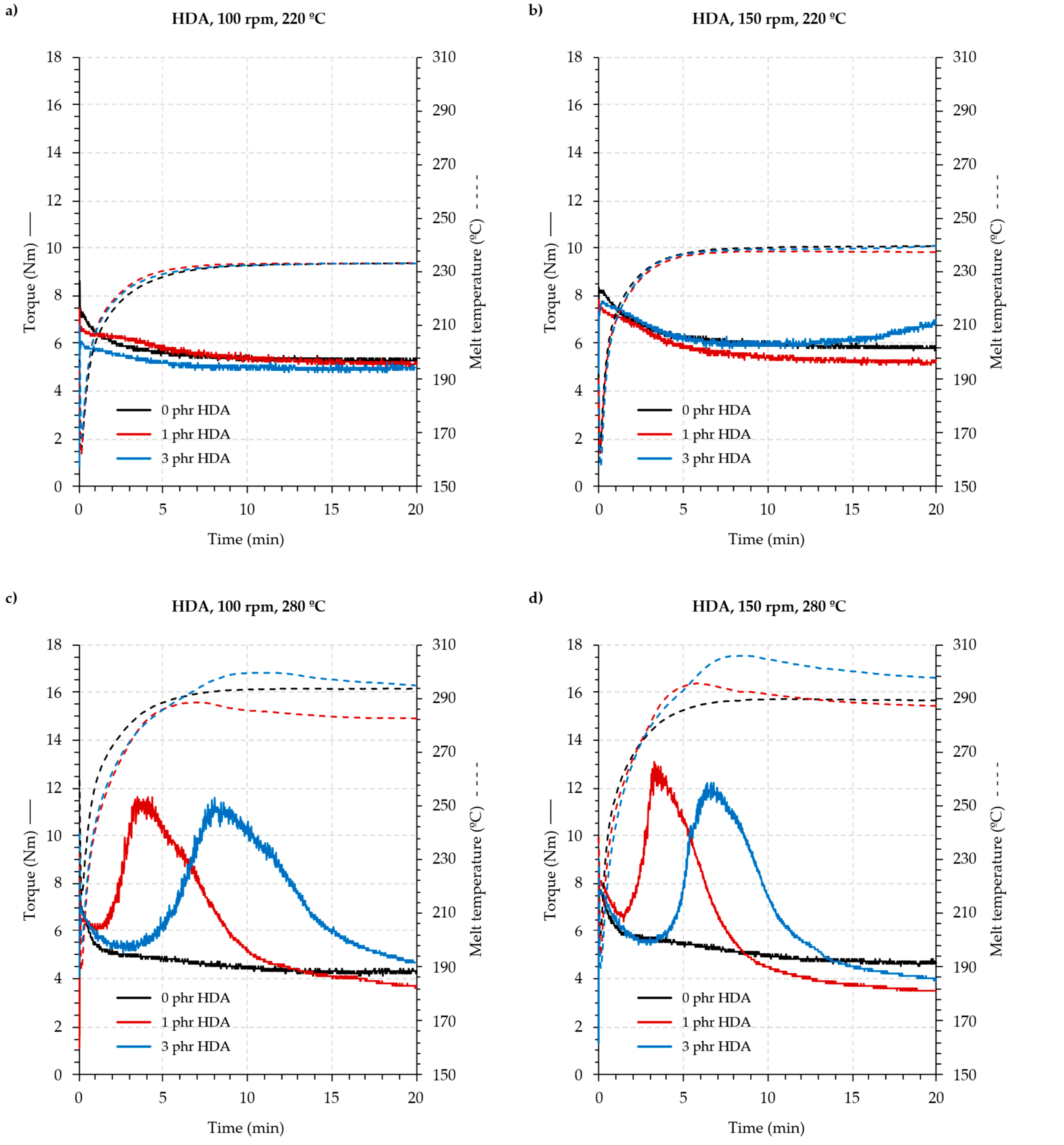

Figure S3. Evolution of torque and melt temperature over time, measured on the laboratory batch mixer devulcanization trials with HDA, at 220 ºC and 280 ºC, at 100 (a and c) and 150 (b and d) rpm, respectively.
